# Pupillary Responses and Vital Signs in Hypoglycemic Patients with Impaired Consciousness During Prehospital Care: A Retrospective Observational Study

**DOI:** 10.3390/diagnostics15121487

**Published:** 2025-06-11

**Authors:** Junko Yamaguchi, Kosaku Kinoshita, Umefumi Iguchi, Tsukasa Kuwana

**Affiliations:** Division of Emergency and Critical Care Medicine, Department of Acute Medicine, Nihon University School of Medicine, 30-1 Oyaguchi Kamimachi, Itabashi-ku, Tokyo 173-8610, Japan; kinoshita.kosaku@nihon-u.ac.jp (K.K.); iguchi.umefumi@nihon-u.ac.jp (U.I.); kuwana.tsukasa@nihon-u.ac.jp (T.K.)

**Keywords:** hypoglycemia, impaired consciousness, prehospital emergency care, autonomic abnormalities, pupil response

## Abstract

**Background/Objectives:** Impaired consciousness has various causes. One such cause includes hypoglycemia, which may be symptomatic or asymptomatic and is associated with high mortality. Autonomic abnormalities are also common in hypoglycemic patients. Early detection is critical for improving prognosis. In this study, we evaluated changes in vital signs and pupillary responses before and after glucose administration in patients with hypoglycemia managed in a prehospital emergency setting. **Methods**: This retrospective observational study included 583 adult patients from the Tokyo Fire Department database. All patients were suspected by emergency medical technicians (EMTs) to have hypoglycemia-related impaired consciousness and showed improved consciousness after receiving intravenous glucose infusion at the scene. Vital signs, level of consciousness, and pupillary responses were assessed before and after glucose administration. **Results**: The mean patient age was 58.9 years, and approximately 90% had comorbid diabetes mellitus. Tachypnea was common at the scene, with 27% showing tachycardia, while blood pressure remained normal. Miosis and abnormal pupillary light reflexes were observed in 68% and 84% of cases, respectively. Anisocoria occurred in 7.6% of the patients. After glucose administration, both abnormal reflexes and anisocoria significantly decreased (both *p* < 0.0001). Although vital signs did not consistently reflect autonomic responses, changes in pupillary findings were prominent. **Conclusions**: Altered pupillary responses are common in hypoglycemic coma. Findings such as miosis and anisocoria can result from various causes, including central nervous system disorders and cholinergic toxicity; thus, careful differential diagnosis is essential. Normal blood pressure may help to distinguish hypoglycemic coma during prehospital care.

## 1. Introduction

Diseases that result in impaired consciousness, such as central nervous system (CNS) and metabolic disorders, are often life-threatening and significantly affect a patient’s quality of life [1]. Severe hypoglycemia is one such condition that leads to impaired consciousness, and early detection and intervention are essential to prevent serious sequelae [2]. Due to these risks, when hypoglycemia is suspected, unconscious patients are administered an intravenous glucose solution by emergency medical technicians (EMTs).

In Japan, during the prehospital stage, EMTs usually assess the severity of illness using protocols established by the regional Medical Control Committee. For patients with impaired consciousness suspected of requiring emergency glucose administration, EMTs measure blood glucose levels using a blood glucose meter and assess the level of consciousness, blood pressure (BP), respiratory rate (RR), heart rate (HR), pupil size, and pupillary light reflex before and after glucose administration. Altered consciousness can result from various causes, including CNS disorders, poisoning, infections, and metabolic abnormalities. Among these, hypoglycemic coma is a relatively rare cause of altered consciousness [3,4]. The frequency of metabolic disorders, including hypoglycemic coma, among coma etiologies is reported to be low at approximately 5% [1].

In the prehospital setting, impaired consciousness may hinder EMTs from obtaining an accurate medical history, including diabetes status, making it difficult to suspect hypoglycemic coma. Additionally, asymptomatic hypoglycemia is a concern, with a relatively high incidence and reported associations with autonomic nervous system abnormalities [5].

This study aimed to investigate whether it is possible to differentiate hypoglycemic coma from other causes of impaired consciousness by evaluating changes in vital signs and pupillary findings in patients with suspected hypoglycemic coma. This was achieved through a retrospective analysis of data from 583 patients who received intravenous glucose in prehospital care according to protocols for suspected hypoglycemic coma.

## 2. Materials and Methods

This single-center observational study was conducted from 1 January 2014 to 31 December 2017. Prehospital data were obtained from the Tokyo Fire Department’s prehospital transportation record database covering the Tokyo metropolitan area. This study was approved by the Ethics Committee of the Nihon University School of Medicine (approval No. 2021-09). Due to the retrospective nature of the study, the requirement for informed consent was waived.

Eligible patients were adults aged >15 years who were transported to hospitals by EMTs in an ambulance and suspected of having hypoglycemia with impaired consciousness. Patients were included if, at the prehospital stage, they were treated with intravenous glucose infusion therapy in accordance with established protocols for suspected hypoglycemia. The EMTs initially assessed the patients by measuring their vital signs and levels of consciousness. Based on the severity of the condition, glucose infusion was administered according to the prehospital protocol (Supplementary Figure S1). EMTs administered intravenous glucose, particularly when altered consciousness was suspected to be caused by hypoglycemia.

In Japan, the Japan Coma Scale (JCS), introduced in 1974, is used to evaluate consciousness levels in prehospital settings [6,7]. This scale allows for a quick and simple assessment based on eye-opening responses. It is known as the 3-3-9 method and is widely used in Japan, especially in emergency prehospital care, where rapid evaluation is essential. The JCS is divided into three major categories: alert, single-digit, two-digit, and three-digit codes. Each digit category was further divided into three subcategories (1, 2, and 3 for single-digit codes; 10, 20, and 30 for two-digit codes; and 100, 200, and 300 for three-digit codes). Additionally, three special conditions—restlessness, incontinence, and apathy—were recorded. The JCS, which consists of nine grades, is widely used to evaluate patient consciousness.

Patients with impaired consciousness suspected to be due to hypoglycemia and classified as JCS-II-10 or higher were considered suitable for intravenous glucose administration under the paramedic blood glucose infusion criteria in Japan. However, because the JCS is not a continuous numerical scale, it poses challenges for statistical analysis compared to scales such as the Glasgow Coma Scale (GCS). Therefore, we considered it necessary to convert JCS categories into a numerical format suitable for statistical evaluation.

Yumoto et al. validated the JCS for assessing in-hospital mortality in trauma cases [7]. In our study, the JCS data were converted to Glasgow Coma Scale (GCS) scores using a validated conversion table to facilitate statistical analysis [8] (Table 1). To assess the impact of converting JCS to a numeric scale, we also analyzed pre- vs. post-treatment changes using the raw JCS categories (I-1, I-2, …, III-300) as an ordinal variable. We assigned them integer codes 1–9 and applied a Wilcoxon signed-rank test for paired ordinal data, reporting median JCS category before and after glucose, the Z-statistic, *p*-value, and the shift in category distribution.

**Figure 1:**
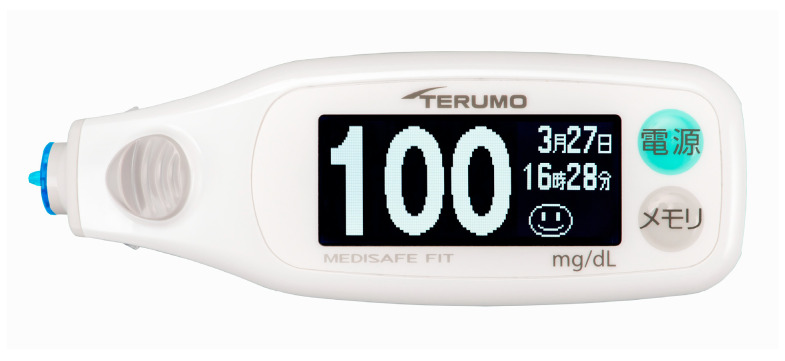
The Medisafe Fit^®^ blood glucose meter (Terumo Corporation, Japan) image using EMT at the prehospital scene for suspected hypoglycemic state with impaired consciousness.

Patients with a JCS code of 10 or more or a blood glucose level <50 mg/dL were eligible. Prior to glucose infusion, blood glucose levels were measured using a portable glucometer (Figure 1).

Patients meeting these criteria received an intravenous infusion of 40 mL of 50% glucose solution. Vital signs and levels of consciousness were recorded at 2 min intervals after glucose administration. In addition, pupil size and pupillary light reflex responses were documented in this study.

**Figure 2:**
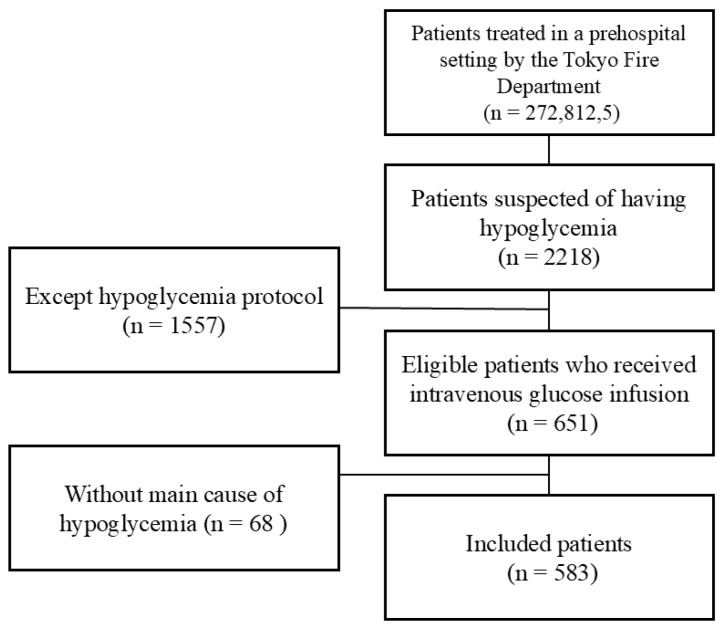
The Consolidated Standards of Reporting Trials (CONSORT) diagram shows patient inclusion in this study.

**Figure 3:**
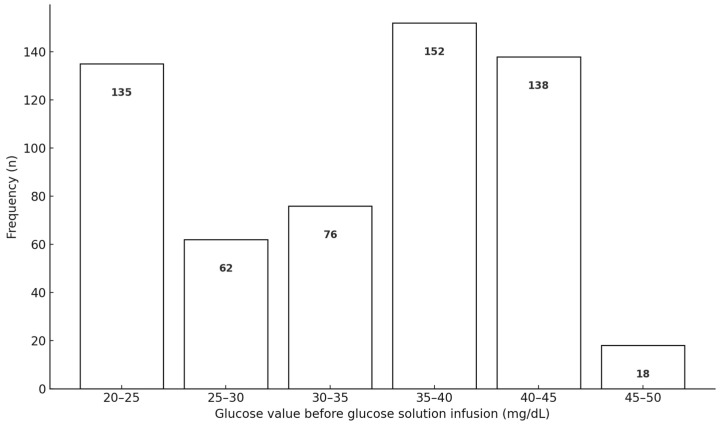
Blood glucose levels prior to intravenous glucose infusion.

Two coauthors with clinical experience in managing hypoglycemic comas reviewed the medical records of 651 patients. Of these, 68 cases that did not meet the protocol criteria were excluded. The final analysis included 583 patients suspected of having hypoglycemic comas who received intravenous glucose infusion (Figure 2). Because portable glucometers cannot accurately measure blood glucose levels below 20 mg/dL, the glucose level was presumed to be 20 mg/dL in such cases (Figure 3).

Demographic information, including age, sex, and the presence of preexisting diabetes, was recorded. Vital signs were evaluated before and after glucose administration. Electrocardiography (ECG) monitoring was also performed during prehospital care.

All statistical analyses were conducted using SPSS version 25 (IBM Statistics, Chicago, IL, USA) and JMP (Version 14.2, SAS Institute, Cary, NC, USA). Data are presented as median (interquartile range [IQR]) or number of cases (%). Continuous variables were compared using Wilcoxon signed-rank tests, and categorical variables were compared using the chi-square test. *p*-value < 0.05 was considered statistically significant.

## 3. Results

### 3.1. Consciousness Level and Vital Signs

The average age of the patients was 58.9 years, and 75.1% were male. Among these patients, 90.1% had diabetes mellitus; however, the diabetes mellitus status of the remaining patients could not be determined. The glucose levels before glucose infusion are shown in Figure 3. The average blood glucose level was 32.9 (standard deviation [SD], 8.41 [min, 20; max, 46] mg/dL).

The levels of consciousness (as indicated by the GCS score calculated using a validated conversion table from JCS), pupillary responses, and ECG results before and after intravenous glucose infusion are summarized in Table 2. Patients’ levels of consciousness improved after glucose administration. However, because the JCS is not a continuous numerical scale, statistical analysis of changes in consciousness levels before and after glucose administration cannot be conducted in the same manner as with the GCS. To ensure our numeric transformation did not bias results, we also treated the nine JCS grades as an ordinal scale (coded 1–9) and reran the Wilcoxon signed-rank test on pre- vs. post-treatment JCS. The median JCS improved from 5 to 2 (Z = −20.92, *p* < 0.001, the effect size was r = 0.268, indicating a moderate effect), mirroring the findings from the converted scores and indicating minimal impact of the transformation. Sinus tachycardia was observed in 27.1% of the patients. The median systolic BP was 138 mmHg (IQR: 124–154), diastolic BP was 132 mmHg (IQR: 120–150), and HR was 84 beats/min (IQR: 78–102), all within normal ranges. In contrast, the median RR was elevated at 24 counts/min (IQR, 18–24), indicating tachypnea.

**Figure 4:**
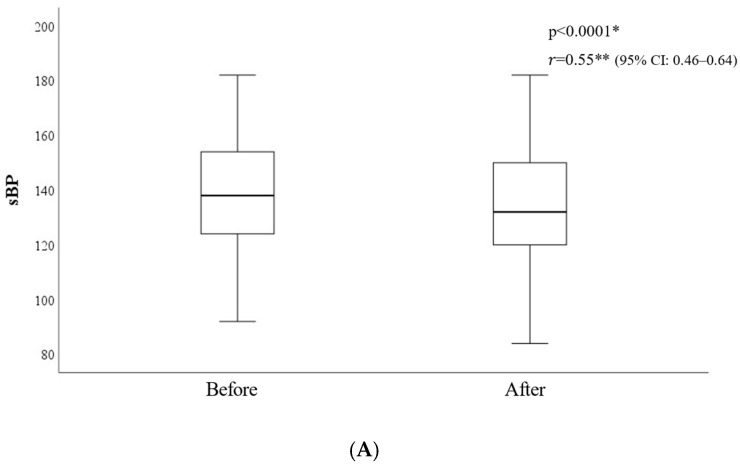

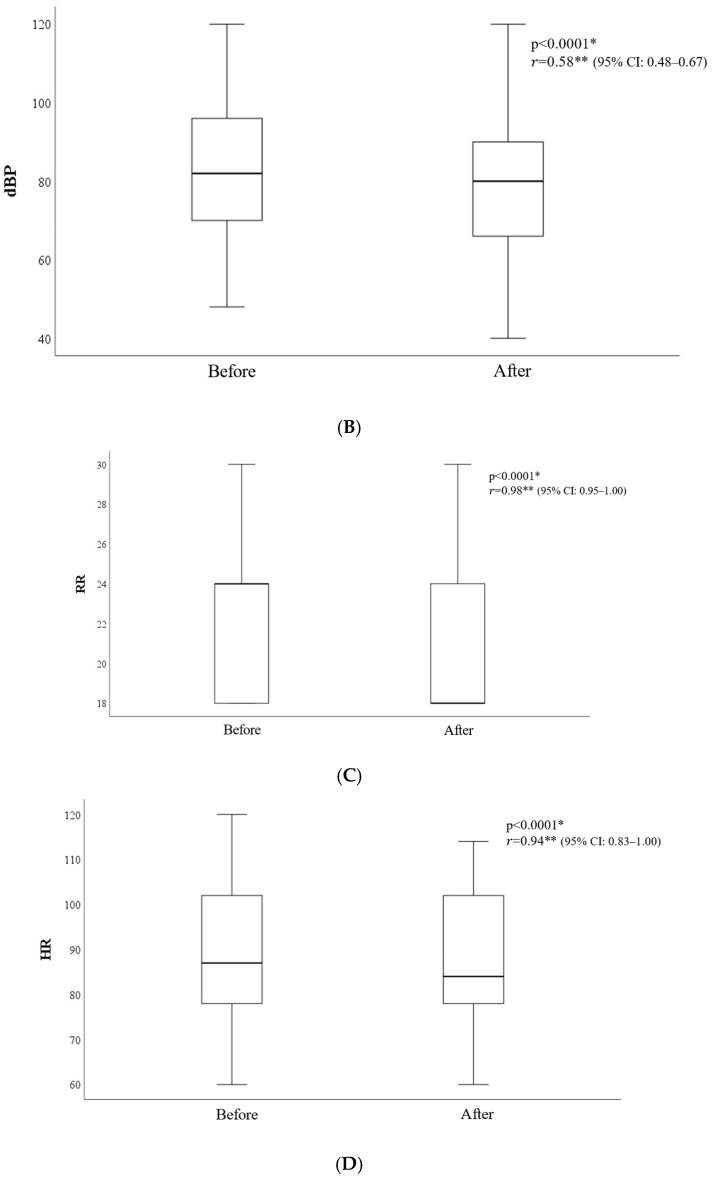
(**A**). Changes in systolic blood pressure before and after glucose administration; * Wilcoxon signed-rank test; ** The effect size *r* for the Wilcoxon signed-rank test was calculated using the formula *r* = Z/√n; Before, Before glucose administration; After, After glucose administration; sBP, systolic blood pressure, CI: Confidence Interval; The median systolic blood pressure significantly decreased after glucose administration (Z = 10.14, *p* < 0.0001, r = 0.55, 95% CI: 0.46–0.64), indicating a medium-to-large effect size. (**B**). Changes in diastolic blood pressure before and after glucose administration; * Wilcoxon signed-rank test; ** The effect size *r* for the Wilcoxon signed-rank test was calculated using the formula *r*= Z/√n; Before, Before glucose administration; After, After glucose administration; dBP, diastolic blood pressure, CI: Confidence Interval; The median diastolic blood pressure significantly decreased after glucose administration (Z = 9.74, *p* < 0.0001, r = 0.58, 95% CI: 0.48–0.67), indicating a large effect size. (**C**). Changes in Respiratory Rate before and after glucose administration; * Wilcoxon signed-rank test; ** The effect size *r* for the Wilcoxon signed-rank test was calculated using the formula *r* = Z/√n; Before, Before glucose administration; After, After glucose administration; RR, respiratory rate; CI: Confidence Interval; The median respiratory rate significantly decreased after glucose administration (Z = 9.01, *p* < 0.0001, r = 0.98, 95% CI: 0.95–1.00), indicating a large effect size. (**D**). Changes in heart rate before and after glucose administration; * Wilcoxon signed-rank test; ** The effect size *r* for the Wilcoxon signed-rank test was calculated using the formula *r* = Z/√n; Before, Before glucose administration; After, After glucose administration; HR, heart rate; CI, Confidence Interval; The median heart rate significantly decreased after glucose administration (Z = 6.01, *p* < 0.0001, r = 0.94, 95% CI: 0.83–1.00), indicating a large effect size.

After glucose infusion, the BP, RR, and HR significantly decreased (*p* < 0.0001) (Figure 4).

### 3.2. Pupil Response

The median pupil size was 2 mm (IQR:2,3) both before and after intravenous glucose infusion and abnormalities in the pupillary light reflex were observed in all patients. Although pupil size and light reflex responses improved significantly following glucose administration, most patients continued to exhibit small pupils and persistent light reflex abnormalities.

Among the 583 patients, 67.5% presented with miosis, and 7.6% exhibited anisocoria. The incidence of anisocoria significantly decreased after glucose infusion (*p* < 0.0001).

## 4. Discussion

Diseases that cause impaired consciousness, including CNS disorders, metabolic abnormalities, and intoxication, are often critical and require early intervention [3,4,9]. Severe hypoglycemia has been reported to cause cognitive impairment and compromise the function of the central nervous, cardiovascular, and cerebrovascular systems [10,11,12].

In emergency settings, the classic Whipple triad, blood glucose ≤ 2.8 mmol/L, symptoms of hypoglycemia, and symptom resolution following glucose administration can facilitate the identification of hypoglycemia [13]. However, hypoglycemia unawareness, particularly in diabetic patients, has been well documented and may present without typical symptoms [14]. Hypoglycemic coma is more common in individuals with diabetes and is sometimes associated with abnormal autonomic responses [5]. In our study, approximately 90% of patients had diabetes mellitus. In these individuals, diabetes itself contributes to impaired glucose counter-regulation and hypoglycemia unawareness. Hypoglycemia further impairs glucose homeostasis by lowering the glycemic threshold for sympathetic–adrenal (including epinephrine) and neural responses to falling plasma glucose. Notably, short-term avoidance of hypoglycemia has been shown to improve hypoglycemia awareness in most patients [14], highlighting the importance of early recognition and treatment of hypoglycemic coma.

Several case reports have described pupillary abnormalities associated with hypoglycemia. One reported transient bilateral miosis in a patient with severe insulin-induced hypoglycemia, while another described prolonged reactive miosis following insulin intoxication [15]. In Japan, a case involved a patient in a hypoglycemic coma whose pupils remained constricted despite preserved light reflexes [16]. Another report noted improved pupillary responses after glucose administration [17]. However, none of these studies specifically examined pupillary findings in hypoglycemic coma during prehospital emergency care. Autonomic neuropathy may also contribute to miosis in diabetic patients. Additionally, age-related peripheral nerve dysfunction, often present early in diabetes, may play a role.

Additionally, parasympathetic hyperactivity or sympathetic hypoactivity may be contributing factors [2,18,19]. Nevertheless, the specific characteristics of autonomic nervous system responses, such as vital signs and pupillary responses, in hypoglycemic patients with impaired consciousness during the acute phase remain unclear, and the underlying mechanisms are still not well understood [20]. In our study, most patients presented with tachypnea, and approximately 30% exhibited tachycardia, which is typically regarded as a normal sympathetic response. Nevertheless, the median systolic and diastolic BP and HR remained within the normal ranges.

Few studies have described the vital signs of patients with hypoglycemic coma in prehospital settings or examined how these parameters change following glucose administration. One report on hospitalized diabetic patients with hypoglycemic coma found that blood pressure significantly decreased after glucose administration. In comparisons between patients with type 1 and type 2 diabetes, those with type 1 diabetes showed smaller changes, likely due to autonomic nervous system dysfunction [21].

Mizu et al. reported that systolic BP < 100 mmHg and high body temperature were characteristics of non-hypoglycemic coma. Their study, which compared hypoglycemic and non-hypoglycemic patients with impaired consciousness in prehospital care, supports our findings that the BP of patients suspected of having hypoglycemic coma was normal [22]. A literature review yielded no studies that specifically examined pupillary findings in hypoglycemic comas during prehospital care.

In our study, miosis was frequently observed, and over 80% of the patients had light reflex abnormalities that significantly improved following intravenous glucose infusion. The pupillary diameter is regulated by the autonomic nervous system, which includes the sympathetic and parasympathetic nervous systems. The radial muscle, innervated by the sympathetic system, dilates the pupil, whereas the sphincter muscle, innervated by the parasympathetic fibers of the oculomotor nerve (cranial nerve III) originating from the Edinger–Westphal nucleus, constricts the pupil. These muscles act antagonistically to control iris function [23,24]. Pupil constriction, or miosis, is primarily a parasympathetic effect. The pupillary light reflex is considered to be one of the most critical functions of pupillary dynamics [25].

Tokuda et al. reported the usefulness of bedside pupil evaluation in determining coma etiology using a probabilistic approach. From a statistical standpoint, abnormalities in the pupillary light reflex and anisocoria may be useful indicators of the structural causes of coma in critical situations [26]. In our study, miosis was a common finding, whereas anisocoria was observed in 7.6% of the patients.

Previous reports have noted that metabolic abnormalities, such as hypoglycemia, account for approximately 5% of coma cases [1]. The present results indicate that when evaluating patients with impaired consciousness in a prehospital setting, it is essential to consider hypoglycemia among the differential diagnoses. However, multiple factors can affect pupil size and pupillary responses, necessitating careful clinical evaluation. Pupil diameter is influenced by physiological, pathological, and pharmacological factors. Physiologically, it tends to decrease with age—a phenomenon known as senile miosis [27]. Sex, height, and body weight have also been linked to ocular biometric variations [28]. Environmental illumination, emotional state, mental arousal, and circadian rhythm similarly affect pupil size [29], as does the circadian rhythm [30].

Diseases affecting pupillary responses include central nervous system disorders (e.g., brainstem lesions, optic nerve disease) and autonomic dysfunctions (e.g., Parkinson’s disease, multiple system atrophy). Pupillary abnormalities may also result from trauma or postoperative complications such as posterior synechiae of the iris [31]. Drug-induced miosis or mydriasis can occur as well. Miosis may be caused by cholinergic agents, opioids, antipsychotics, or sedatives, whereas mydriasis can result from anticholinergic or sympathomimetic agents. In our study, anisocoria was observed in 7.6% of patients with hypoglycemia. Pathologic anisocoria may result from conditions causing unilateral miosis or mydriasis [32], including Horner’s syndrome [33], oculomotor nerve palsy, and Adie’s pupil [34]. Although drug-induced pupillary changes are typically bilateral, unilateral exposure to topical agents may cause anisocoria with a preserved light reflex [31]. Physiological anisocoria has been reported in about 13.7% of the population [35]. In addition to hypoglycemia, miosis and abnormal light reflexes may be influenced by diabetes mellitus. Most patients in our study with impaired consciousness due to hypoglycemia had a known diagnosis of diabetes. In a study comparing 36 patients with insulin-dependent diabetes mellitus and 36 age- and sex-matched controls, the diabetic group showed abnormally small pupil diameters in darkness and reduced pupillary oscillations (hippus) under continuous illumination. Reflex responses to light flashes, adjusted for retinal sensitivity, were also diminished, suggesting autonomic neuropathy secondary to diabetes [36].

Autonomic dysfunction of pupillary control has been reported to precede cardiovascular autonomic changes in diabetic patients [25,37]. Some evidence suggests that early-stage type 2 diabetes-related pupillary dysfunction may be reversible with improved glycemic control [38]. However, studies in children with type 1 diabetes indicate that pupillary function is not significantly affected by current blood glucose levels [39].

These findings underscore the multifactorial nature of pupillary abnormalities, indicating they cannot be attributed solely to hypoglycemic coma. In our study, although statistically significant changes were observed in systolic and diastolic blood pressure, heart rate, respiratory rate, and pupillary parameters two minutes after glucose administration compared to pre-administration values (Table 2, Figure 4), individual variability was notable. In several cases, vital signs remained unchanged, and pupillary reflex abnormalities persisted, suggesting the influence of underlying factors such as diabetic autonomic neuropathy.

In our study, miosis was frequently observed, and BP and HR were within normal ranges. These findings suggest that BP and HR abnormalities may serve as indicators of CNS disease or drug intoxication when accompanied by impaired consciousness, whereas normal BP and HR in the presence of miosis may suggest hypoglycemic coma.

This study had several limitations. First, this was an observational study. Only patients with hypoglycemia and impaired consciousness who received intravenous glucose therapy during the prehospital stage were included. If EMTs did not suspect a hypoglycemic coma, blood glucose levels were not measured, and such cases were not included in this study. Obtaining detailed medical history and medication information in prehospital settings is inherently difficult. Additionally, comorbidities and medications that may influence autonomic responses were not considered. Only the initial glucose level was assessed, and post-infusion glucose levels were not measured. Therefore, we cannot exclude the possibility that changes in consciousness, vital signs, and pupil responses were influenced by factors other than glucose administration. Future research should aim to utilize larger datasets to adjust for potential confounding factors and to identify clinically relevant threshold values that can improve the accuracy of hypoglycemia suspicion in prehospital settings. Subsequently, if existing protocols for identifying suspected hypoglycemia can be modified based on these findings and validated through prospective studies, it may become possible to more reliably recognize hypoglycemia-induced impaired consciousness in real-world emergency care scenarios.

## 5. Conclusions

Changes in vital signs and pupillary responses were observed in hypoglycemic patients with impaired consciousness. Pupillary changes, including miosis, abnormal pupillary light reflexes, and anisocoria, were frequently noted. Although tachypnea was observed, BP remained within the normal range. Given that miosis and anisocoria may also be observed in CNS disorders and cholinergic intoxication, careful clinical differentiation is necessary. However, normal BP may serve as a helpful clue for distinguishing hypoglycemic coma in a prehospital setting.

## Figures and Tables

**Table 1:** Convert the Japan Coma Scale (JCS) to the Glasgow Coma Scale (GCS) *.

	Consciousness Level Assessment by JCS	Convert JCS to GCS
0	Alert	15
1. Single-digit (I)	Awake without any stimuli	
	1 Almost fully conscious but not normal	15
	2 Unable to recognize time, place, and person	14
	3 Unable to recall name or date of birth	13
2. Double-digits (II)	Arousable by some stimulus but reverts to the previous state if stimulus stops	
	10 Arousable by being spoken to	12
	20 Arousable by loud voice	12
	30 Arousable only by repeated mechanical stimuli	9
3. Triple-digits (III)	Unarousable by any forceful stimuli	
	100 Unarousable but responds to avoid the stimuli	7
	200 Unarousable but responds with slight movements, including decerebrate or decorticate postures	6
	300 Does not respond at all	3

* reproduced from Nakajima et al. 2023, [8] licensed under CC BY4.0; JCS, Japan Coma Scale; GCS, Glasgow Coma Scale.

**Table 2:** Characteristics of patients with hypoglycemic coma in a prehospital setting (*n* = 583).

	Before	After	*p*-Value	Effect Size ^b^
Level of Consciousness				
Alert	0/583 (0)	0/583 (0)		
JCS 1-digit [Y/N (%)] JCS1 JCS2 JCS3	0/583 (0)	583/583 (100) 485/583 98/583 1/583	<0.001 *	*r* = 0.92
JCS 2-digit [Y/N (%)] JCS10 JCS20 JCS30	431/583 (73.9) 94/431 71/431 266/431	0/583(0)		
JCS3-digit [Y/N (%)] JCS100 JCS200 JCS300	152/583 (26.1) 31/152 121/152 0/152	0/583 (0)		
Median GCS (IQR) ***	9 (7,9)	15 (15,15)	<0.001 *	*r* = 0.88
Pupil responses				
Median pupil size L (IQR) (mm)	2 (2, 3)	3 (3, 3)	<0.0001 *	*r* = 0.81
Median pupil size R (IQR) (mm)	2 (2,3)	3 (3, 3)	<0.0001 *	*r* = 0.81
Existence of miosis L	395/583 (67.8)	126/583 (21.6)	<0.0001 **	V = 0.18
Existence of miosis R	394/582 (67.7)	120/581 (20.7)	<0.0001 **	V = 0.16
Existence of anisocoria [Y/All (%)]	44/582 (7.6)	6/582 (1.0)	<0.0001 **	V = 0.32
Light reflex abnormality (L) [Y/All (%)]	488/583(83.7)	430/582 (73.8)	<0.0001 **	V = 0.21
Light reflex abnormality (R) [Y/All (%)]	491/583(84.2)	438/583(75.1)	<0.0001 **	V = 0.24
ECG monitor				
Sinus tachycardia ^a^ (%) [Y/All (%)]	158/583 (27.1)	130/583 (22.3)	<0.0001 **	V = 0.87

Before: Prior to glucose infusion; After: Following glucose infusion; ^a^ Sinus tachycardia is defined as a heart rate >100 beats per minute with sinus rhythm; ^b^ The effect size *r* for the Wilcoxon signed-rank test was calculated using the formula *r*= Z / √n; V; Cramér’s V; * Wilcoxon signed-rank test; ** Chi-square test; *** Calculated using a validated conversion table from JCS; JCS, Japan Coma Scale; GCS, Glasgow Coma Scale; IQR, interquartile range; L, light; R, right.

## Data Availability

Data supporting the findings of this study are available from the corresponding author, J.Y., upon reasonable request.

## Supplementary Materials

The following supporting information can be downloaded from https://www.mdpi.com/article/10.3390/diagnostics15121487/s1, Figure S1: The protocol for glucose infusion in suspected hypoglycemia.
