# Supplementary figures and images for: Pupillary Responses and Vital Signs in Hypoglycemic Patients with Impaired Consciousness During Prehospital Care: A Retrospective Observational Study
Source: Diagnostics (Basel). 2025 Jun 11;15(12):1487. doi: 10.3390/diagnostics15121487 (PMC12191695; doi:10.3390/diagnostics15121487)

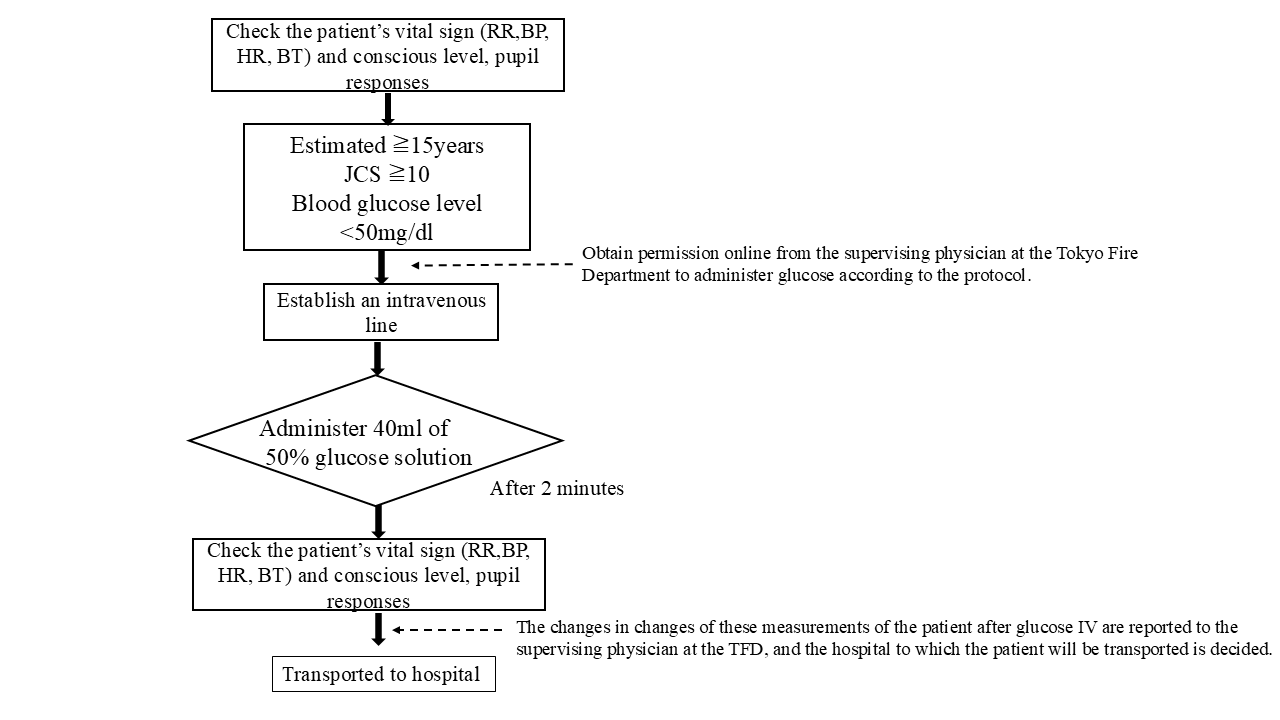

Supplement: Supplementary file 1 [file diagnostics-15-01487-s001.zip › Figure S1.tif]
